# Analysis of Transcriptome and Epitranscriptome in Plants Using PacBio Iso-Seq and Nanopore-Based Direct RNA Sequencing

**DOI:** 10.3389/fgene.2019.00253

**Published:** 2019-03-21

**Authors:** Liangzhen Zhao, Hangxiao Zhang, Markus V. Kohnen, Kasavajhala V. S. K. Prasad, Lianfeng Gu, Anireddy S. N. Reddy

**Affiliations:** ^1^Basic Forestry and Proteomics Research Center, College of Forestry, Fujian Provincial Key Laboratory of Haixia Applied Plant Systems Biology, Fujian Agriculture and Forestry University, Fuzhou, China; ^2^Program in Cell and Molecular Biology, Department of Biology, Colorado State University, Fort Collins, CO, United States

**Keywords:** *SMRT* isoform sequencing, nanopore direct **RNA** sequencing, RNA modification, alternative splicing, alternative polyadenylation, epitranscriptome

## Abstract

Nanopore sequencing from Oxford Nanopore Technologies (ONT) and Pacific BioSciences (PacBio) single-molecule real-time (SMRT) long-read isoform sequencing (Iso-Seq) are revolutionizing the way transcriptomes are analyzed. These methods offer many advantages over most widely used high-throughput short-read RNA sequencing (RNA-Seq) approaches and allow a comprehensive analysis of transcriptomes in identifying full-length splice isoforms and several other post-transcriptional events. In addition, direct RNA-Seq provides valuable information about RNA modifications, which are lost during the PCR amplification step in other methods. Here, we present a comprehensive summary of important applications of these technologies in plants, including identification of complex alternative splicing (AS), full-length splice variants, fusion transcripts, and alternative polyadenylation (APA) events. Furthermore, we discuss the impact of the newly developed nanopore direct RNA-Seq in advancing epitranscriptome research in plants. Additionally, we summarize computational tools for identifying and quantifying full-length isoforms and other co/post-transcriptional events and discussed some of the limitations with these methods. Sequencing of transcriptomes using these new single-molecule long-read methods will unravel many aspects of transcriptome complexity in unprecedented ways as compared to previous short-read sequencing approaches. Analysis of plant transcriptomes with these new powerful methods that require minimum sample processing is likely to become the norm and is expected to uncover novel co/post-transcriptional gene regulatory mechanisms that control biological outcomes during plant development and in response to various stresses.

## Introduction

Analysis of transcriptomes, which represent the activity of genes in the genome, is vital for understanding the relationship between genotype and phenotype. The dynamics and complexity of transcriptome regulate all aspects of plant growth, development, and responses to various external biotic and abiotic cues. Different methods such as expressed sequence tag (EST) sequencing (Wu et al., 2002), serial analysis of gene expression (SAGE) (Matsumura et al., 1999), DNA microarray (Hihara et al., 2001), and recently RNA sequencing (RNA-Seq) using next-generation sequencing (NGS) technologies (Mortazavi et al., 2008) have been developed to analyze transcriptomes. Since 2005, second-generation short-read sequencing platforms quickly replaced first-generation Sanger sequencing technology for various high-throughput applications due to lower costs and greater sequencing depth (Sedlazeck et al., 2018). However, the read length is the major limitation in second-generation short-read sequencing, which made it harder to analyze several aspects of co/post-transcriptional processing events. To overcome this limitation, in the past few years, researchers are sequencing full-length transcripts mostly using two platforms, Pacific BioSciences (PacBio) (Rhoads and Au, 2015) and Oxford Nanopore Technologies (ONT) (Bayega et al., 2018), which are referred to as “third” and “fourth” generation sequencing technologies, respectively (Slatko et al., 2018). These two platforms increased read length considerably as compared to other NGS methods and can, therefore, be used to address a larger variety of research questions. Single-molecule real-time (SMRT) isoform sequencing (Iso-Seq) using PacBio platform captures the full length of transcripts (Gonzalez-Garay, 2016) and thereby presents easier and more accurate ways for different applications, such as gene annotation (Zhao et al., 2018), isoform identification (Abdel-Ghany et al., 2016; Wang T. et al., 2017), identification of fusion transcripts (Weirather et al., 2015), and long non-coding RNA (lncRNA) discovery (Li et al., 2016). Here, we discuss applications and broader utility of PacBio and ONT in transcriptome studies. Recently developed direct RNA-Seq using nanopore can avoid amplification biases (Garalde et al., 2018). Furthermore, this technology has the potential to provide a complete view of RNA modifications such as N^6^-methyladenosine, 5-methylcytidine, and 5-hydroxylmethylcytidine (Li X. et al., 2017), which are collectively referred to as the “epitranscriptome.”

Parts of the core algorithm for PacBio and ONT long-read analyses are similar to short-read analysis strategies used in second-generation sequencing approaches. Nevertheless, specific new bioinformatics tools have been designed for several of the applications, which have not been part of second-generation sequencing pipelines. These tools are needed to provide greater flexibility to achieve different goals as well as to address new issues, such as higher error rates and low throughput. We present currently available bioinformatics methods for PacBio and ONT read analysis, including reads-of-interest (ROI) extraction, error correction (Au et al., 2012), mapping (Wu and Watanabe, 2005), isoform clustering (Fu et al., 2012), and identification of multiple transcript isoforms (Abdel-Ghany et al., 2016). Improvements in these new methods and computational pipelines will expand the landscape of transcriptome complexity at the transcript isoform and epitranscriptome level with higher throughput and higher accuracy. Here, we discussed PacBio Iso-Seq and ONT direct RNA-Seq methodologies, the current status of bioinformatics tools used to analyze the long-reads and highlighted various applications of these methods.

## Library Preparation and Extraction of Read-Of-Insert From Pacbio Iso-Seq

Generally, high-quality RNA is poly(A) selected to construct PacBio long-read sequencing libraries using, e.g., Clontech SMARTer PCR kit (Ramsköld et al., 2012; Li et al., 2016). The length of sequencing reads is dependent on the quality of RNA and generation of full-length cDNAs. To enrich for full-length cDNAs in the library, cap-dependent linker ligation method has been used (Cartolano et al., 2016). Alternatively, full-length RNAs can be enriched by combining poly(A)^+^ RNA selection with capturing of 5′ capped mRNAs using a cap-binding protein (Blower et al., 2013). Full-length mRNA is then used for first-strand cDNA synthesis with oligo (dT) primer followed by second-strand cDNA synthesis with a size selection of full-length cDNA in several different sizes (Xu et al., 2015). With the new Sequel system, cDNAs can be sequenced without size selection. By ligating hairpin adaptors to double-stranded cDNA, SMRTbell^TM^ libraries are generated which can be subsequently sequenced on either the RSII or Sequel platform (Xu et al., 2017). Comparison of 5′ ends with annotated transcript start sites shown that this protocol enables full-length cDNA sequencing with little loss of 5′ or 3′ ends (Ramsköld et al., 2012).

At present, PacBio offers two fourth-generation sequencers: the RSII was the first commercially available sequencing instrument and the recently improved Sequel device provides much higher throughput (up to 20 Gb per SMRT Cell). PacBio’s sequencing strategy is based on the usage of zero-mode waveguide (ZMW) technology, which consists of tiny nano-wells initially described in 2003 (Levene et al., 2003). The ZMWs allow the immobilization of sequencing templates through the interaction with the sequencing engine, a polymerase enzyme complex, which is affixed at the bottom of ZMWs (Rhoads and Au, 2015). Then the incorporation of fluorescent-labeled DNA bases emits fluorescent signals that are captured by a detector in real time (McCarthy, 2010). Hairpin adaptors that are added to both ends of double-stranded DNA during library preparation generate a closed circular DNA template, which could be repeatedly traversed by long lifetime polymerase to improve the accuracy. In this way, PacBio platform could generate multiple subreads including adapter sequences in a single ZMW and yield a continuous long read (CLR), which can generate more accurate circular consensus sequence (CCS) reads (Weirather et al., 2017).

Subsequently, the RSII system and the Sequel system store the base-call data and associated quality metrics in HDF5 and BAM files format, respectively. The bax2bam tool can convert HDF5 file format into BAM format^1^.

**Figure 1:**
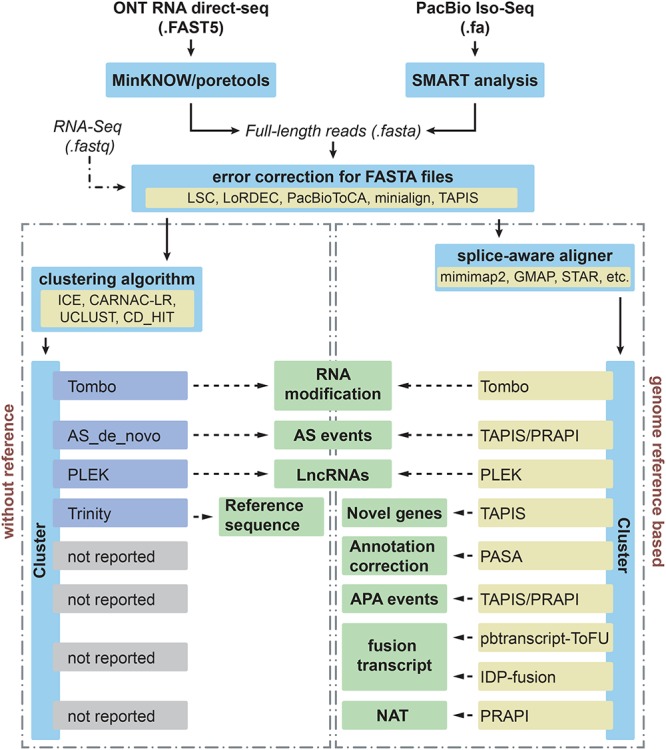
Different applications and bioinformatics solutions for PacBio Iso-Seq and Nanopore direct RNA sequencing in plants. Iso-seq and direct RNA sequencing can be performed using the SMRT analysis from PacBio and MinKNOW from ONT, respectively. Then these long reads with FASTA format will undergo error correction step before downstream analysis. In order to deal with different applications (middle boxes), computational tools used to process and analyze long-reads for each application are indicated for species without and with reference genomes in left and right boxes, respectively.

The SMRT Analysis module from SMRT Link from PacBio is adopted for obtaining effective subreads (Figure 1). Then extraction of ROI for each ZMW is the second step in PacBio Iso-Seq bioinformatics analysis workflow. This step is performed with the SMRT Link pipeline, which includes steps for trimming adapters and generating CCSs. Then ROIs are cleaned of polyA/T tails, primers, artificial concatemers, and transcript strand direction is identified (Bayega et al., 2018). ToFu Pacbio pipeline from SMRT Analysis package can be used to search for sequencing adapters for extracting ROI and full-length non-chimeric (FLNC) reads (Wang T. et al., 2017; Xu et al., 2017). Afterward, the FLNC reads, which contain both 5′ and 3′ primers and poly-A tail, can be analyzed using iterative clustering for error correction (ICE) to build consensus clusters to improve consensus accuracy. Subsequently, PacBio RS II and Sequel use Quiver and Arrow to polish consensus sequences, respectively (Bayega et al., 2018).

## Library Construction and Base-Calling for Nanopore Direct RNA Sequencing

The starter pack for direct RNA-Seq costs only $1000 (pricing as of January 2019), which includes one MinION sequencer, two flow cells, one sequencing kit, and a wash kit^2^. Compared to NGS or PacBio, the MinION is portable (weighs 90 g), real-time, long-read, and low-cost device. It is also possible to use the SMARTer protocol for full-length cDNA synthesis (Ramsköld et al., 2012), which includes end-repair, dA-Tailing, and adapter ligation. However, this kind of library construction will remove all RNA modification information during double-strand cDNA synthesis. The Nanopore direct RNA library construction workflow uses poly(dT) adapter and SuperScript III Reverse Transcriptase to generate RNA–DNA hybrids, which are subsequently ligated to nanopore sequencing adapters using T4 DNA ligase prior to sequencing. Then Agencourt RNAClean XP magnetic beads are used to purify RNA–DNA duplexes^3^. After estimating the sample concentration, the Nanopore direct RNA library can be loaded into flow cells using MinION, GridION, or PromethION sequencer. Compared to the MinION, the GridION and PromethION sequencers provide higher throughput. The motor protein pulls the 3′ end of the RNA strand inside the nanopore channel (Bayega et al., 2018). Then changes in the ionic current are detected at each pore by a sensor.

Prior to sequencing, the computer hardware should be checked to meet the minimum requirement. The minimal requirements for MinION are: CPU: i7 or Xeon with 4+ cores, memory: 16 GB RAM, storage: 1 TB internal SSD, ports: USB3^4^. Any computer with above minimal requirements can run a MinION without deterioration of performance during sequencing. Desktop or laptop computer with a MinKNOW and EPI2ME Desktop Agent installation provided by Oxford Nanopore and Metrichor Ltd., respectively, need to be connected with MinION (Figure 1). MinKNOW controls the MinION device, tests hardware, checks flow cells, and performs sequencing runs. EPI2ME further analyzes the raw electrical signals generated and stores in FAST5 files, which serve as input for Metrichor for base-calling. Then FASTQ and FASTA sequences can be extracted from FAST5 files using poretools (Loman and Quinlan, 2014). However, detection tools to identify base modifications are currently not available. The majority of the applications using Nanopore direct RNA-Seq have been focused on mammals. At present, Nanopore direct RNA-Seq has not been reported for studies on plants. However, it is anticipated that more and more laboratories will use this tool to study RNA modifications in plants.

## Long-Read Preprocessing: Error Correction, Mapping, and Clustering of Long-Reads

Although the length of PacBio and ONT reads is longer than NGS, one common concern regarding these technologies is high error rates (Koren et al., 2012). Thus, it is necessary to reduce the error rate before subsequent utilization. At present, correcting PacBio and ONT reads fall into three distinct categories: hybrid error correction strategy, self-correction method, and reference-based error correction.

Hybrid error correction strategy uses short reads from NGS to correct long reads. LSC (Au et al., 2012), LoRDEC (Salmela and Rivals, 2014), and PacBioToCA (Koren et al., 2012) are three widely used methods for error correction (Figure 1). Unlike LSC and PacBioToCA, LoRDEC avoids mapping of short reads by building short reads De Bruijn graph (DBG) of order *k* and threads the long reads through this short reads DBG to correct. Thus LoRDEC requires less time/memory and less disk space (Salmela and Rivals, 2014). Recently, Nanocorr was developed specifically to correct Nanopore long reads using high-quality short reads (Goodwin et al., 2015).

Alternatively, self-correction software is distinct from the above hybrid error correction strategy, which depends on short-reads. Long-read multiple aligner (LoRMA) is one of the methods for error correction that relies only on long reads (Salmela et al., 2016). Compared to another self-correction method PacBio corrected reads (PBcR) algorithm (Koren et al., 2012), LoRMA achieved higher throughput and lower error rate. However, self-correction method needs a high coverage in order to obtain accurate correction, which limits its application.

The third method provides reference-based error correction during alignment of long reads to reference genome and some tools that do this type of error correction are minimap2 (Li, 2018) and minialign^5^. These are fast and accurate alignment tools for PacBio and Nanopore long reads with high insertion and deletion error rate. Transcriptome Analysis Pipeline from Isoform Sequencing (TAPIS) (Abdel-Ghany et al., 2016) also performs reference-based error correction. In addition to minimap2, GMAP (Wu and Watanabe, 2005) and STAR (Dobin et al., 2013) are two splice-aware aligners, which can be used for mapping full-length reads to reference genome for downstream analysis. However, GMAP and STAR do not perform error correction during mapping. In addition to canonical splice sites, GMAP and STAR capture non-canonical splice sites, hence should be cautious during downstream AS analysis. Compared to GMAP, minimap2 is more consistent with existing annotation and works well with noisy reads (Li, 2018).

Highly expressed genes could generate multiple identical isoforms, which would take more time for downstream processing/analyses and are hard to visualize without collapsing redundant reads. Clustering step could group full-length reads into a cluster, which is a necessary step to further improve quality and identify unique splicing isoforms. After mapping Iso-Seq to reference genome, Cupcake ToFU could be used to collapse redundant isoforms and obtain unique isoforms^6^. The majority of clustering strategies used for species without reference genome have been developed for ESTs, which appeared before the age of PacBio and ONT. Clustering programs designed for ESTs, such as UCLUST (Edgar, 2010) and CD-HIT (Fu et al., 2012), are widely used to group and collapse redundant sequences. However, these methods were not designed for full-length sequences with high error rates as compared to ESTs or short reads from NGS. At present, there are two *de novo* algorithms for clustering of long reads by genes: the ICE algorithm (Gordon et al., 2015) can cluster FLNC reads from PacBio sequencing to generate consensus isoforms and the CARNAC-LR algorithm designed for ONT long-read sequencing data (Marchet et al., 2018). After collapsing the redundant isoforms, the read count information for expression levels would be lost. If expression level analysis needs to be performed, one can go back and retrieve the read counts from the original sequencing files.

## Applications and Bioinformatics Tools for Iso-Seq and Nanopore Direct RNA Sequencing in Plants

At present, PacBio and ONT deep sequencing are increasingly used for genome annotation, identification of co/post-transcriptional events and fusion transcripts. Recently, several studies collected and reanalyzed long reads from Iso-Seq into comprehensive databases such as Plant ISOform sequencing database (PISO) (Feng et al., 2019) and ISOdb (Xie et al., 2018). ISOdb and PISO deposited 8 and 19 species, respectively. Since the new technology has a higher resolution than second-generation sequencing and detects modified RNA bases, additional aspects of transcriptional and post-transcriptional regulation can be studied more comprehensively. Therefore, we highlight bioinformatics solutions and various applications that are difficult to investigate using NGS.

## *De Novo* Genome Annotation, New Locus Identification, and Gene Model Correction

For species without an available reference genome, such as *Drynaria roosii* (Sun et al., 2018) and *Asparagus officinalis* (Kakrana et al., 2018), Iso-Seq was successfully used recently to capture the complete and full-length transcriptome. Due to the longer reads from PacBio and ONT, Iso-Seq has proven to be more advantageous in resolving many complex features in transcriptomes when compared to short-read RNA-Seq, which depends on software for reconstructing transcript sequences (Haas et al., 2013; Steijger et al., 2013). Thus, one key advantage of long-reads from PacBio and ONT was to accurately infer gene models by generating full-length transcripts without further assembly, which is challenging for complex isoforms (Gordon et al., 2015). The utility of long-read transcripts in inferring gene models has been reported in medicinal herb *Panax ginseng* (Jo et al., 2017; Kim et al., 2018),allohexaploid wheat (Clavijo et al., 2017), bread wheat (Cartolano et al., 2016), sugar beet (Minoche et al., 2015), the coffee bean (Cheng et al., 2017), and Para rubber tree (Pootakham et al., 2017). Full-length transcripts generated by Iso-Seq are ideal for improving gene model prediction and identification of novel genes, which do not map to annotated gene loci. For example, recent studies revealed 2171 novel genes in *Sorghum bicolor* (Abdel-Ghany et al., 2016), 8091 in *Phyllostachys edulis* (Wang T. et al., 2017), and 3026 in *Triticum aestivum* (Gordon et al., 2015). Also in *Populus trichocarpa* (Filichkin et al., 2018), allopolyploid cotton (Wang et al., 2018), and *Populus* “*Nanlin 895*” (Chao Q. et al., 2018), 15,087, 13,551, and 1575 novel transcribed regions, respectively, were recently identified. In addition to isoform and new locus identification, Iso-Seq has been used to refine gene models in *Vitis vinifera* cv. Cabernet Sauvignon (Minio et al., 2018) and allopolyploid cotton (Wang et al., 2018). Furthermore, recent studies corrected 178 and 2241 annotated genes, which covered more than one transcript assemblies in *S. bicolor* (Abdel-Ghany et al., 2016) and *P. edulis* (Wang T. et al., 2017), respectively. Program to Assemble Spliced Alignments (PASA) is one bioinformatics tool that corrects such gene annotations (Haas et al., 2008). Recently, long-read annotation (LoReAn) pipeline used a combination of PacBio SMRT or MinION long-reads and other information such as protein evidence for gene annotation (Cook et al., 2019).

## Characterization of Alternative Transcription Initiation, Alternative Polyadenylation, and Alternative Splicing

Alternative transcription initiation (ATI), alternative cleavage and alternative polyadenylation (APA), and alternative splicing (AS) events are three major processes that contribute to transcriptome diversity. AS of precursor mRNAs (pre-mRNAs) can potentially increase the number of protein isoforms produced from multiexon genes and regulate gene expression through multiple mechanisms such as altered translational efficiency of splice isoforms, non-sense-mediate decay, and miRNA-medicated mRNA degradation (Reddy et al., 2013). Though individual AS events can be quantified and annotated using NGS with great accuracy, it is hard to deduce full-length splicing isoforms that contain a combination of these individual AS events (Steijger et al., 2013). Long-read sequencing provides the possibility to obtain full-length sequences and thus identify complex splice isoforms, which are hard to detect and reconstruct by NGS. Iso-Seq has allowed identification of over 110,00 non-redundant isoforms in *Zea mays* (Wang et al., 2016), >42,000 in *P. edulis* (Wang T. et al., 2017), and >16,000 in *Salvia miltiorrhiza* (Xu et al., 2015). Additionally, Iso-Seq identified 29,730 novel isoforms in *Trifolium pratense* L., 2501 new alternative transcripts in *V. vinifera* cv. Cabernet Sauvignon (Minio et al., 2018), and over 11,000 novel splice isoforms in *S. bicolor* L. Moench (Abdel-Ghany et al., 2016). For 35.74% of the unigenes of bermudagrass, three or more distinct isoforms were identified using Iso-Seq (Zhang B. et al., 2018). In the wild strawberry *Fragaria vesca*, Iso-Seq revealed that pre-mRNAs from ∼58% of multiexon genes are alternatively spliced (Li Y. et al., 2017).

In addition to the full-length isoform detection, AS events can be classified into five different types: retained intron (RI), skipped exon (SE), alternative 5′ splicing site (A5SS), alternative 3′ splicing site (A3SS), and mutually exclusive exons (Shen et al., 2014). In addition to above five common categories, many other complex types, such as alternative position, i.e., alternative 3′ and 5′ site (Wang and Brendel, 2006), AS and transcriptional initiation (ASTI) (Nagasaki et al., 2006) alternative first exons (Chen et al., 2007), and composite patterns (Wang and Rio, 2018), can occur. Although NGS can detect these AS events, long reads from PacBio and ONT provide an advantage on detecting AS events because long-read sequencing could avoid any possible issues during transcriptome reconstruction. For example, Iso-Seq revealed 10,053, 172,743, 133,229, and 21,154 AS events in *S. bicolor* (Abdel-Ghany et al., 2016), *Z. mays* (Wang et al., 2016), allopolyploid cotton (Wang et al., 2018), and *P. edulis* (Wang T. et al., 2017), respectively.

Alternative polyadenylation has multiple regulatory roles in RNA transportation, localization, stability, and translation by producing isoforms with different 3′ cleavage sites, which generates transcript diversity and complexity (Tilgner et al., 2015; Abdel-Ghany et al., 2016; Wang T. et al., 2017). For APA identification using NGS, Poly(A) Site Sequencing (PAS-Seq) libraries can be constructed using degenerate nucleotides in combination with oligo(T) primers (Shepard et al., 2011; Zhang et al., 2015). Internal priming issue was defined as cDNA primers hybridizing to internal continuous As instead of the actual poly(A) tail (Beaudoing et al., 2000). If six continuous As or more than seven As existed in a 10 nt window, it was internal priming candidate (Tian et al., 2005). PAS-Seq based on NGS methods could not avoid the internal priming because internal A-rich sequences could prime the oligo(dT) (Nam et al., 2002; Sherstnev et al., 2012). Both Iso-Seq and Nanopore direct RNA-Seq methods could avoid internal priming. Using Iso-Seq, 7700 genes containing two or more polyadenylation sites have recently been detected in *S. bicolor* (Abdel-Ghany et al., 2016). In allopolyploid cotton, 6935 genes have at least five poly(A) sites (Wang et al., 2018). At present, quantification analysis of APA still depends on NGS due to the low sequence depth of Iso-Seq and Nanopore direct RNA-Seq. A recent study in *P. edulis* used a method that combined NGS with Iso-Seq to identify 1224 differential APA sites (Wang T. et al., 2017). In the future, it is expected that both Iso-Seq and Nanopore direct RNA-Seq can be used for quantification analysis once the throughput increases.

Alternative transcription initiation is another key mechanism to generate diverse transcripts (Tanaka et al., 2009). Alternative usage of transcription start sites attracted little attention in plants as compared to the studies on AS and APA. Paired-end analysis of transcription start sites (PEAT) strategy, which requires complex library construction, following NGS has been used for monitoring global transcription start site usage (Ni et al., 2010). Using the PEAT protocol, millions of transcription start sites that fall into three categories have been identified in Arabidopsis roots (Morton et al., 2014). Since PacBio Iso-Seq and Nanopore direct RNA-Seq can sequence full-length transcripts from 5′ ends to polyadenylated tails, it would be a perfect tool to detect ATI.

For traditional RNA-Seq, the identification of the major AS events, including exon skipping events, intron retention, alternative 5′ donor, and alternative 3′ donor usage is quite simple by using several tools, including rMATS (Shen et al., 2014), JUM (Wang and Rio, 2018), PASA pipeline (Campbell et al., 2006), and ASTALAVISTA (Foissac and Sammeth, 2007). For the analysis of post-transcriptional regulation based on long-read sequencing, TAPIS pipeline (Abdel-Ghany et al., 2016) and PRAPI (Gao et al., 2017) are two main bioinformatics tools that use Iso-Seq reads to identify AS and APA (Figure 1). In addition, PRAPI (Gao et al., 2017) can also identify several other events/processes, such as ATI, and production of circular RNAs (circRNAs).

## Identification of Fusion Transcripts

Fusion transcripts are the result of a trans-splicing event (Li et al., 2008) that joins two separately encoded pre-RNAs into one transcript. Fusion transcripts have been identified in diverse plant species (Zhang et al., 2010; Wang et al., 2016). Paired-end RNA-Seq datasets based on NGS have been successfully analyzed for fusion transcript (Maher et al., 2009). Recently, Iso-Seq provided a more reliable way to identify fusion transcripts. In total, 1430 fusion transcripts had been detected in *Z. mays* using Iso-Seq (Wang et al., 2016). Furthermore, 3762 and 222 fusion transcripts were identified in *T. pratense* L (Chao Y. et al., 2018) and allopolyploid cotton (Wang et al., 2018), respectively.

The standard for fusion transcript identification is based on the simple idea that two or more fragments from one transcript can be mapped to several loci (Wang et al., 2016). Multiple fusion transcript detection algorithms based on NGS have been developed (Liu S. et al., 2015). However, these algorithms were specially designed for paired-end RNA-Seq data. PacBio pbtranscript-ToFU package provides a script to detect fusion transcripts (fusion_finder.py)^7^, which is specially designed for reads from Iso-Seq. Isoform Detection and Prediction (IDP) fusion (Figure 1) also presents another algorithm to detect fusion events using both PacBio long-read sequencing and NGS (Weirather et al., 2015).

## lncRNA Identification

Long ncRNAs are defined as RNAs with more than 200 nt and have no discernable coding potential (Jin et al., 2013). In plants, lncRNAs can be generated from intergenic, intronic, or coding regions and play an important role in gene regulation (Wang and Chekanova, 2017). The majority of lncRNAs are polyadenylated in plants, thus RNA-Seq on Illumina platforms can also detect the expression of lncRNAs. However, recent studies showed that lncRNAs undergo complex post-transcriptional regulation (Liu J. et al., 2015). Thus, full-length sequencing provides a great advantage in identifying gene model of lncRNAs. Recently, several studies reported the identification of lncRNAs using Iso-Seq in plants. For example, PacBio Iso-Seq revealed 1187 and 4333 lncRNAs in poplar “*Nanlin 895*” (Chao Q. et al., 2018) and *T. pratense* L. (Chao Y. et al., 2018), respectively. These studies suggested that Iso-Seq is a well-suited method for identification of lncRNAs. GreeeNC and CANTATAdb are two resources to search for sequence homology of lncRNAs from long reads, which have been reported in *P. edulis* (Wang T. et al., 2017). Also, long reads containing sequence homology to miRNAs could also be regarded as non-coding RNA, as has been reported in *S. bicolor* (Abdel-Ghany et al., 2016). In *Z. mays*, lncRNAs were identified using PLEK, a classification model trained on known high-confidence lncRNAs (Wang et al., 2016).

## Natural Antisense Transcripts Identification

Natural antisense transcripts (NATs) including head-to-head, tail-to-tail, and fully overlapping types have been shown to function in transcriptional and post-transcriptional gene regulation (Faghihi and Wahlestedt, 2009). In total, 932 *cis*-NATs were identified using a strand-specific PacBio SMRT dataset by performing pair-wise comparisons of overlapping coordinates from oppositely oriented full-length transcripts (Zhang H. et al., 2018). Furthermore, PRAPI was developed to identify NAT based on PacBio/ONT long reads (Gao et al., 2017). At the same time, PRAPI can also quantify the expression of NAT by combining NGS reads using strand-specific library construction (Figure 1).

## Analysis of Long-Reads in the Absence of a Reference Genome

Due to recent developments in long-read sequencing, more and more genome sequencing studies are using long-read sequencing platforms to obtain longer reads than N50, such as *de novo* assembling of grass *Oropetium thomaeum* (VanBuren et al., 2015), sunflower (Badouin et al., 2017), and citrus (Wang X. et al., 2017). However, there are still many species without available genome sequences. Thus, it will be valuable to develop reference-free analyses for transcription annotation using Trinity (Haas et al., 2013) and other tools for post-transcriptional analysis. Recent studies have shown that it is feasible to reconstruct full-length transcript models for species without a reference genome, such as *Astragalus membranaceus* (Li J. et al., 2017), *Arabidopsis pumila* (Yang et al., 2018), and *Zanthoxylum bungeanum* Maxim (Tian et al., 2018) using long reads.

Recently, AS_*de*_*novo*^8^ has reported AS identification based on Iso-Seq without reference genomes (Liu et al., 2017). The basic idea originated from searching for the deletion or insertion in the clustering units (Ner-Gaon et al., 2007; Zhou et al., 2011; Wu et al., 2014; Liu et al., 2017). Thus, clustering long reads from PacBio Iso-Seq or ONT should be the first step before AS identification. Several clustering programs, such as the widely used CD-HIT, can be used for this analysis (Fu et al., 2012). Recently, one clustering approach designed for Oxford Nanopore long reads has been released (Marchet et al., 2018). After the clustering step, all-vs-all BLAT comparison can be used for the identification of insertion or deletion segmentation caused by AS events (Liu et al., 2017). Hybrid sequencing and map finding (HySeMaFi) combined PacBio Iso-Seq and NGS to identify splicing and quantify the isoforms abundance (Ning et al., 2017). AStrap adopted machine-learning model to identify AS events by integrating more than 500 assembled features (Ji et al., 2018).

## The Application of Nanopore Direct RNA Sequencing

**Figure 2:**
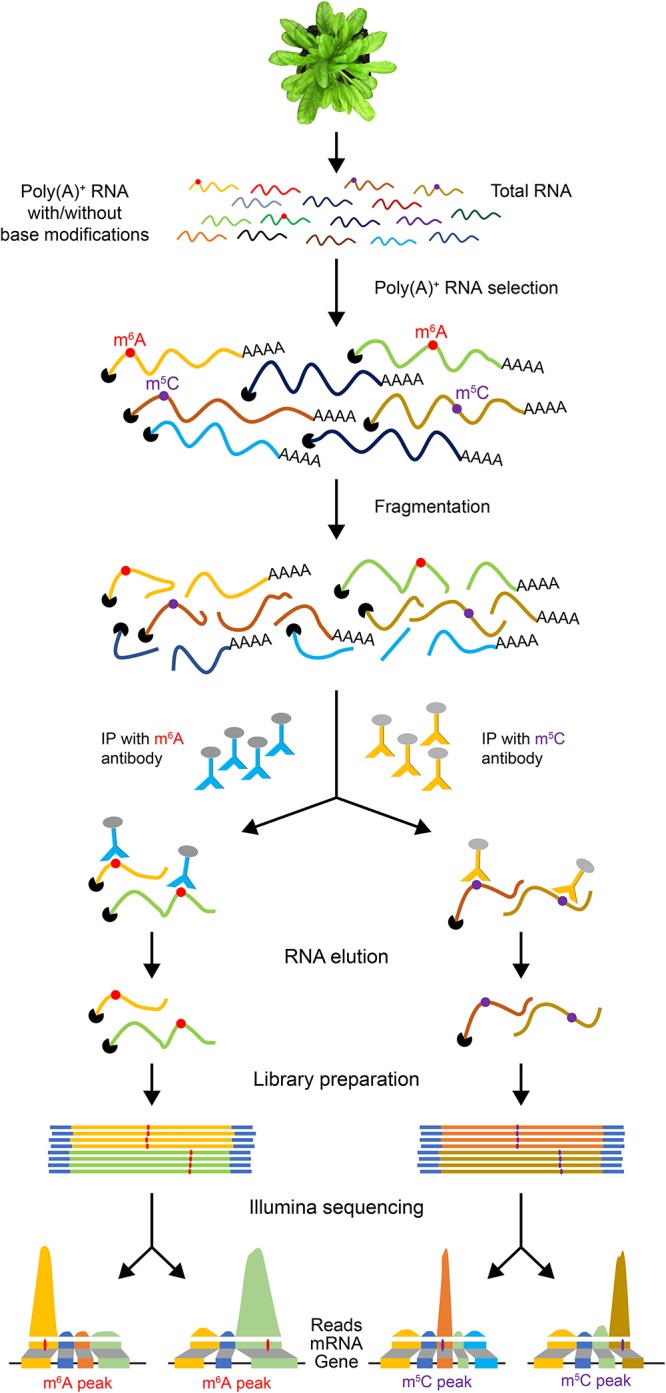
An illustration of epitranscriptome analysis using antibodies to identify RNAs with base modifications. Poly(A)^+^ mRNA is used for RNA immunoprecipitation with antibodies specific to a base modification (e.g., m^6^A or m^5^C). The IP’ed RNA is then used to generate a cDNA library for high-throughput sequencing. The reads are then aligned to the reference genome.

Since full-length native RNA-Seq (nRNA-Seq) of ONT provides multiple benefits compared to NGS, this method has been applied for detecting viral transcriptomes (Moldován et al., 2018a), 16S rRNA base modifications (Smith et al., 2017), viral pathogen (Depledge et al., 2018), and identification of artifactual splice isoforms during reverse transcription due to the template switching (Moldován et al., 2018b). Finally, a significant advantage of direct RNA-Seq is that it allows detection of co/post-transcriptional base modifications in RNA since it does not require reverse transcription and PCR amplification steps. Many reversible chemical modifications of bases occur in mRNAs, which are collectively referred to as the “epitranscriptome” (Gilbert et al., 2016). These covalent reversible chemical modifications of nucleotides regulate many aspects of gene expression. Recent studies indicate that epitranscriptomic modifications are key players in regulating pre-mRNA splicing, nuclear export, mRNA stability and localization, and translation efficiency (Gilbert et al., 2016; Xiao et al., 2016; Roundtree et al., 2017; Slobodin et al., 2017) and also several developmental processes in plants (Fray and Simpson, 2015; Vandivier and Gregory, 2018). There is no simple high-throughput tool to detect mRNA modifications and their dynamics in plants. A widely used method for transcriptome-wide analysis of RNA modifications is challenging as it requires specific antibodies for each modification. These antibodies are then used to precipitate RNA with modifications, which is then subjected to high-throughput sequencing (Figure 2). This method has been used to identify transcriptome-wide m^6^A localization and abundance in animals (Dominissini et al., 2012; Meyer et al., 2012). In *Arabidopsis thaliana*, a transcriptome-wide 6-methyladenine (m^6^A) and 5-methylcytosine (m^5^C) profiles were reported using the m^6^A- or m^5^C-targeted antibodies, respectively, for RNA immunoprecipitation (RIP) followed by high-throughput sequencing (m^6^A-Seq/m^5^C-Seq) (Luo et al., 2014; Cui et al., 2017). This RIP-Seq approach has several limitations including the need for specific antibody for each modification. It is also time-consuming and laborious. Furthermore, it is difficult to obtain a sufficient amount of immunoprecipitated RNA. More importantly, this method does not provide the precise location of the modified base. Recently, it has been shown that RNA modifications can be detected using Oxford Nanopore direct RNA-Seq (Garalde et al., 2018).

**Figure 3:**
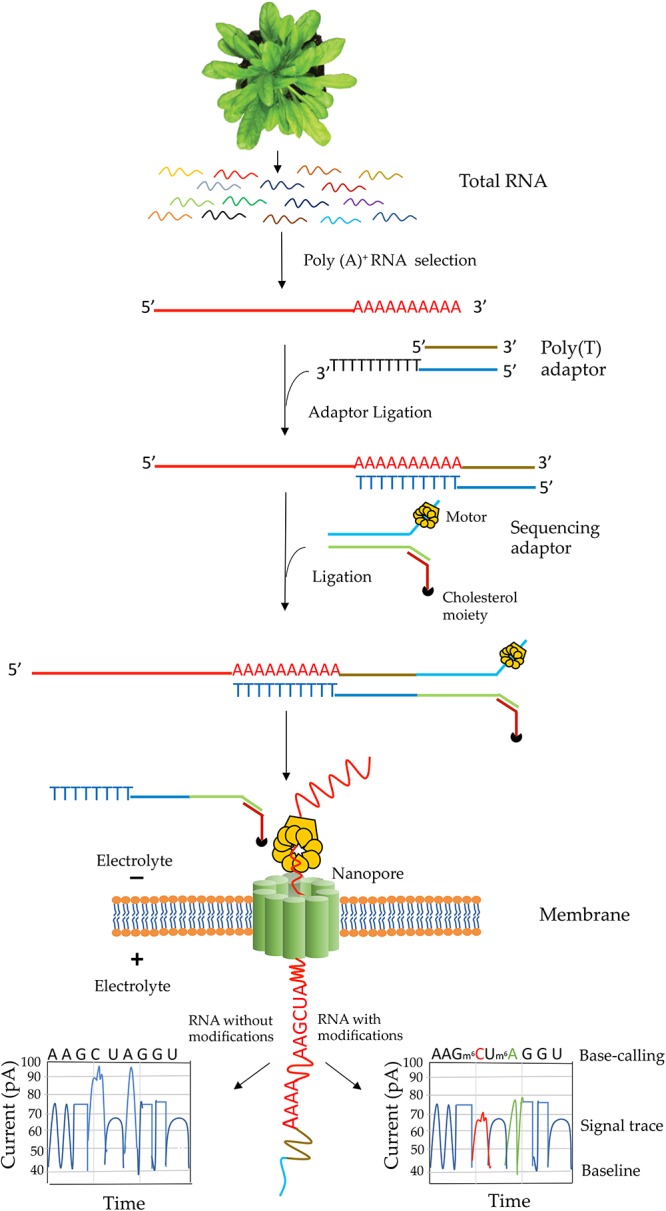
Schematic illustration of direct RNA sequencing using the Oxford Nanopore Technology. Poly(A)+ mRNA from total RNA is isolated, then a poly(T) adaptor and a sequencing adaptor with a motor enzyme are added to the 3′ end of poly(A)+ mRNA. It is then subject to sequencing on a membrane with thousands of nanopore channels, each of which is coupled to ammeters that measure current passing through the pore. The motor enzyme interacts with a nanopore on an electrically resistant synthetic membrane and the RNA strand is fed through the nanopore. A voltage across the membrane is applied and as the RNA moves through the nanopore nucleotide bases cause a characteristic change in current through the pore that is unique to each normal and modified base. The current output is then used in base-calling. An example of current output when RNA with (right box) or without modified RNA bases (left box) move through a pore is shown.

The library construction protocol for direct RNA-Seq was designed for poly(A) transcripts (Garalde et al., 2018). Steps involved in native RNA-Seq are illustrated in Figure 3. Although single-stranded RNA is depicted in this figure, RNA-DNA hybrid can be used for direct RNA-Seq where only the RNA strand in the hybrid is sequenced. The use of RNA-DNA hybrids may alleviate some issues associated with RNA secondary structures and improve sequence throughput and quality (Garalde et al., 2018). In characterizing the yeast transcriptome using direct RNA-Seq, single-stranded RNA was used (Garalde et al., 2018) whereas in analyzing the human transcriptome, RNA–DNA hybrids were used (Workman et al., 2018). Transcripts without poly(A) tail can also be sequenced by enzymatically adding a 3′ poly (A) tail. One of the limitations for direct RNA-Seq is about the truncated reads. Studies in both pseudorabies virus (Moldován et al., 2018b) and *Saccharomyces cerevisiae* (Jenjaroenpun et al., 2018) revealed truncated reads, especially missing nucleotides at the 5′ end of the transcripts. It was speculated that it might be due to the premature release of the sequencing transcripts by the motor protein (Moldován et al., 2018b). However, longer transcripts over 5 kb could be generated using direct RNA-Seq (Jenjaroenpun et al., 2018). Thus, the motor protein might not be the major reason for the truncated reads. Another limitation is that at present bioinformatics tools for identification of RNA modification are rare. Tombo is the only reported tool to identify modified nucleotides from ONT (Stoiber et al., 2016). Also, base-calling algorithms for most RNA modifications are yet to be developed. Recently, soybean (*Glycine max*) seed transcriptome has been sequenced using MinION sequencing. However, this study adopted cDNA sequencing method, which could not be used for characterization of RNA modifications (Fleming et al., 2018). So far, only two direct RNA-sequencing studies – one with yeast poly(A)^+^ RNA (Garalde et al., 2018) and one with human poly(*A)^+^ RNA(*Workman et al., 2018) – have been performed with eukaryotic mRNAs. Interestingly, native sequencing of human poly(A)^+^ RNA uncovered a large number of novel isoforms (over 65% of all detected isoforms are novel) (Workman et al., 2018). The authors of the human transcriptome study were able to assess poly(A)^+^ length, allele-specific expression, base modifications (N^6^-methyladenine and inosine) in mRNA from direct RNA-Seq data (Workman et al., 2018).

## Future Directions

From the Iso-Seq library construction step, it becomes apparent that the RNA modification information will be removed. Thus, common Iso-Seq libraries cannot be used for detecting RNA modification. Beside direct RNA-Seq, the PacBio reads from genome sequencing without any PCR amplification step can be used to detect DNA methylation marks, such as m^6^A, m^5^C, 5-hydroxymethylcytosine (Flusberg et al., 2010; Fang et al., 2012), and 4-methylcytosine (4mC) (Ye et al., 2016), respectively. Bisulfite sequencing (BS-Seq) using NGS can also detect m^5^C in a genome-wide manner (Krueger et al., 2012). However, long reads without PCR amplification provide new opportunities to detect additional modifications, which present distinct kinetic profiles and cannot be detected using NGS technologies. In *A. thaliana*, global profiling of m^6^A residues has been investigated using this method at single-nucleotide resolution (Liang et al., 2018). ONT sequencing can detect native genomic methylation, which has been reported in *Escherichia coli* (Rand et al., 2017) and humans (Simpson et al., 2017). It can be expected that both PacBio and ONT with enough coverage can replace present methylation detecting methods, such as bisulfite-treated DNA following NGS for m^5^C identification (Frommer et al., 1992). By using a reverse transcriptase, instead of DNA polymerase, in ZMWs, cDNA synthesis has been observed in real time (Saletore et al., 2012). Furthermore, the presence of a modified (e.g., m^6^A) in RNA has been shown to alter the kinetics of nucleotide incorporation at the modified site. Based on this, it was suggested that by monitoring cDNA synthesis in real time in ZMWs modifications in RNA can be identified using the altered kinetic signature (Saletore et al., 2012).

Previous studies have shown that it is difficult to reconstruct splice isoforms and quantify differential expression of isoforms using short reads obtained with second-generation sequencing (Steijger et al., 2013; Kratz and Carninci, 2014). In comparison with Illumina, the read length is the great advantage in Iso-Seq cDNA transcript sequencing and Oxford Nanopore direct RNA-Seq, which can capture entire transcripts (Wang et al., 2016). Comparison of the gene expression between Illumina datasets and MinION revealed high correlation coefficient (Seki et al., 2018), which suggests that MinION is a useful platform to calculate expression level of transcripts by read count, or relative abundance of an RNA as transcripts per million transcripts (TPM) (Marinov, 2017). Indeed, single-molecule long-read sequencing in maize revealed tissue-specific isoforms (Wang et al., 2016). These new technologies provide great strengths and new avenues to explore complex transcriptomes. A combination of different techniques can offer solutions to overcome weaknesses of NGS and PacBio/ONT (Rhoads and Au, 2015). At present, IDP (Au et al., 2013) was developed to use long reads for identification of complex transcript structure and next-generation short reads for quantification. This hybrid method can solve the limitation for both technologies. A recent study showed a high correlation between ONT and Illumina on quantifying gene expression (Byrne et al., 2017). With improvements in sequencers (from MinION, GridION to PromethION), Oxford Nanopore direct RNA-Seq with sufficient throughput and accuracy can possibly be used to perform quantitative analyses of full-length isoforms on a whole transcriptome level.

## Author Contributions

LZ, HZ, MK, KP, LG, and AR wrote, discussed, and edited the manuscript.

## Conflict of Interest Statement

The authors declare that the research was conducted in the absence of any commercial or financial relationships that could be construed as a potential conflict of interest.

## Footnotes

1 https://github.com/PacificBiosciences/PacBioFileFormats/wiki/BAM-recipes

2 https://nanoporetech.com

3 https://nanoporetech.com

4 https://community.nanoporetech.com

5 https://github.com/ocxtal/minialign

6 https://github.com/Magdoll/cDNA_Cupcake

7 https://github.com/PacificBiosciences

8 https://github.com/liuxiaoxian/IsoSeq_AS_de_novo
